# A review of the therapeutic potential of histone deacetylase inhibitors in rhabdomyosarcoma

**DOI:** 10.3389/fonc.2023.1244035

**Published:** 2023-08-18

**Authors:** Omar Selim, Clara Song, Amy Kumar, Rebecca Phelan, Arun Singh, Noah Federman

**Affiliations:** ^1^ Clinical and Translational Science Institute, University of California, Los Angeles, CA, United States; ^2^ Department of Medicine, David Geffen School of Medicine, University of California, Los Angeles, CA, United States; ^3^ Department of Pediatrics, David Geffen School of Medicine, University of California, Los Angeles, CA, United States; ^4^ Department of Orthopaedic Surgery, David Geffen School of Medicine, University of California, Los Angeles, CA, United States

**Keywords:** rhabdomyosarcoma, histone deacetylase inhibitors, pediatric cancer, clinical trials, mutations

## Abstract

This review aims to summarize the putative role of histone deacetylases (HDACs) in rhabdomyosarcoma (RMS) and the effects of HDAC inhibitors (HDACi) on RMS by elucidating and highlighting known oncogenic pathways, mechanisms of resistance, and the synergistic potential of histone deacetylase inhibitors. We searched two databases (PubMed and Google Scholar) for the keywords “Rhabdomyosarcoma, histone deacetylase, histone deacetylase inhibitors.” We excluded three publications that did not permit access to the full text to review and those that focus exclusively on pleiomorphic RMS in adults. Forty-seven papers met the inclusion criteria. This review highlights that HDACi induce cytotoxicity, cell-cycle arrest, and oxidative stress in RMS cells. Ultimately, HDACi have been shown to increase apoptosis and the cessation of embryonal and alveolar RMS proliferation *in vivo* and *in vitro*, both synergistically and on its own. HDACi contain potent therapeutic potential against RMS. This review discusses the significant findings and the biological mechanisms behind the anti-cancer effects of HDACi. Additionally, this review highlights important clinical trials assessing the efficacy of HDACi in sarcomas.

## Introduction

Rhabdomyosarcoma (RMS) is an aggressive soft tissue cancer, accounting for most soft tissue sarcomas in the pediatric population. Although RMS can present across all age groups, this malignancy is most prevalent in children between the ages of 0 and 14 (1). RMS accounts for 3.5% of all pediatric cancers and 50% of all soft tissue sarcomas in children between 0 and 14 years of age with an incidence rate of approximately 4.5 cases for every 1 million children in the United States under the age of 20 (2, 3).

The two most common subtypes of RMS are embryonal RMS (ERMS) and alveolar RMS (ARMS). ERMS is commonly found in the head, neck, and reproductive organs and accounts for 60% of RMS diagnoses. ARMS is the more aggressive form of RMS with a poorer prognosis and is found in the limbs and trunk of the body, accounting for approximately 20% of RMS cases (4).

The phenotypic features of RMS resemble the biological characteristics and behavior of embryonic skeletal muscle, suggesting that the pathogenesis of the disease is linked to errors in the differentiation of myoblasts in embryos (4). This disease is typically marked by rhabdomyoblasts, which are early forms of mesenchymal cells that experience incomplete myogenesis in the differentiation process to become skeletal muscle cells (5). As such, RMS primarily exists in skeletal muscle, but the disease can also be found in hollow organs, including the bladder, bile ducts, and uterus (6).

Current treatments for RMS involve multimodal approaches that involve a combination of cytotoxic chemotherapies in addition to radiation and surgery, as appropriate. Although recent advances in RMS research have led to significant improvements in the prognosis for patients, the 5-year survival rate for this disease remains at about 70% (7). However, the survival rate also depends on the tumor type and, more importantly, translocation status, which will be discussed in greater detail in subsequent sections. The outcomes for patients with recurrent, refractory and/or metastatic RMS are dismal, with 5-year event-free survival at less than 20% and little incremental improvements in outcome in the past four decades (7). Thus, there is a significant need for novel approaches to treat RMS.

Histone deacetylases (HDACs) are proteins that serve as epigenetic regulatory factors that remove acetyl groups from lysine residues of histones, resulting in more repressive chromatin structures (8). As such, HDACs can regulate cellular processes by modifying chromatin conformation to decrease gene expression. In general, acetylation leads to a decondensed chromatin state, favoring transcription. As such, HDACs reverse the open configuration. These epigenetic factors have been associated with genes related to tumorigenesis, including regulation of apoptosis, cell growth, and differentiation (8).

Due to HDACs having various mechanisms for different types of cancers, histone deacetylase inhibitors (HDACi) have been investigated across several different clinical indications in oncology as a novel anticancer treatment in combination with other drugs or radiotherapy. HDACi are small molecule inhibitors of HDAC activity, retaining decondensed chromatin states that promote gene expression. At present, the FDA has approved four HDACi for cutaneous/peripheral T-cell lymphoma (9, 10). Additionally, there are ongoing studies utilizing HDACi as a cancer treatment for solid tumors, blood cancers, lung cancers, thyroid cancers, inflammatory breast cancer, glioblastoma, melanoma, and myeloma (11–13). More pertinent to this review, two clinical trials are currently investigating RMS (**Table 1**).

**Table 1:** HDACi for the treatment of sarcomas under clinical investigation.

HDACi	Class	Dose	Frequency	Sarcoma	Phase	Status	Sponsor	NCT#
Belinostat	I, II, IV	600-1000 mg/m^2^	5 days/week	Soft Tissue Sarcoma	I/II	Completed	Onxeo	NCT00878800
Chidamide	I	30mg	b.i.w.	Sarcoma	II	Recruiting	Sun Yat-sen University	NCT04025931
Entinostat	I	1) 2mg/m^2^ 2) 2-12mg/m^2^	q.d	Solid Tumors	I	Completed	National Cancer Institute	NCT00020579
Mocetinostat	I, IV	40mg	t.i.w	RMS	I	Recruiting	Mirati Therapeutics Inc.	NCT04299113
Panobinostat	I, II, IV	40mg	b.i.w.	Soft Tissue Sarcoma	II	Completed	Centre Leon Berard	NCT01136499
Panobinostat	I, II, IV	15mg	t.i.w	Soft Tissue Sarcoma	I	Completed	Novartis Pharmaceuticals	NCT01005797
Quisinostat	I, II, IV	1) 2-4mg 2) 6-12mg 3) 15-19mg	1) q.d. 2) q.d. 3) t.i.w.	Solid Tumors	I	Completed	Johnson & Johnson Pharmaceutical Research & Development, L.L.C.	NCT00677105
Romidepsin	I	13mg/m^2^	q.w.	Soft Tissue Sarcoma	II	Completed	National Cancer Institute	NCT00112463
Valproic Acid	I, IIa	40mg/kg	5 days/month	Sarcoma/Soft Tissue Sarcoma	I	Completed	Genetech, Inc.	NCT01106872
Vorinostat	I, IIa	Information unknown		RMS	I	Recruiting	Merck & Co.	NCT04308330
Vorinostat	I, IIa	400mg	q.d.	Soft Tissue Sarcoma	II	Completed	Merck Sharp & Dohme LLC	NCT00918489
Vorinostat	I, IIa	400mg	q.d.	Soft Tissue Sarcoma	II	Completed	National Cancer Institute	NCT00937495
Vorinostat	I, IIa	1) 180mg/m^2^ 2) 230mg/m^2^ 3) 300mg/m^2^	5 days/week	Sarcoma	I	Completed	National Cancer Institute	NCT01132911
Vorinostat	I, IIa	1) 300mg 2) 200mg	t.i.w	Sarcoma	I/II	Completed	Merck Sharp & Dohme LLC	NCT01879085
Vorinostat	I, IIa	270mg/m^2^	q.d.	Sarcoma	I/II	Completed	Merck Sharp & Dohme LLC	NCT01294670
Trichostatin A	I, II	No reported clinical trials studying solid tumors to date.						

b.i.w., twice a week; q.d., every day; t.i.w., three times a week; q.w., once a week.

Although the exact mechanisms behind the pathogenesis of the RMS are yet to be elucidated, there is a growing body of evidence that suggests HDACs contribute to the proliferation of RMS (8). As a result, with the improved understanding of the molecular and genetic background of HDACi and ongoing clinical investigation, HDACi may be a promising new class of drugs in the treatment of RMS. In this review, we evaluate the current findings and understanding of the antineoplastic activity of HDACi in RMS.

## Review

### Molecular pathogenesis of rhabdomyosarcoma, translocation status and implications for HDAC inhibitors

As mentioned, RMS can be divided into two major histologic subtypes - embryonal (ERMS) and alveolar (ARMS). However, it is now widely recognized that translocation status rather than histology alone (i.e. embryonal vs. alveolar) is the most important factor for risk stratification and overall prognosis. Whole-genome sequencing of tumor and normal somatic pairs revealed genotypes of RMS that are characterized by the fusion of PAX3 or PAX7 with FOXO1 (PAX-FOXO1) or those without these fusions (14). For ERMS, although it is fusion-negative (FN-RMS), it is often found to have a mutation in Ras signaling and loss of heterozygosity at the 11p15 chromosomal region (4). Regarding fusion-positive (FP-RMS), which is typically ARMS, PAX3 fuses with FOXO1 t(2;13) (q35;q14) to produce PAX3-FOXO1 and t(1;13) (p36;q14) produces PAX7-FOXO1 (1, 4, 15).

Although, generally, ARMS is PAX-FOXO1 FP-RMS and ERMS is FN-RMS, this is not always the case. Roughly only 80% of patients with ARMS are FP-RMS (11). Due to the distinction between FP- and FN- RMS, there are notable variations regarding the types of HDACi that exhibit activity in RMS. Altered susceptibilities to therapeutic approaches, including HDACi, depend on these distinctions as well as the broad spectrum of HDACi, which act on diverse HDAC classes (**Table 2**). Interestingly, FP-RMS cases showed an increased sensitivity to treatments targeting HDACs compared to FN-RMS. For example, the HDACi, valproic acid, in combination with the small molecule inhibitor PKC412 (midostaurin), was shown to downregulate PAX-FOXO1 activity, demonstrating antineoplastic activity against ARMS (16).

**Table 2:** HDAC classes and interaction complexes.

Family	HDAC Class	Subclass	Complex Interactions	Protein	HDACi	Cell Compartment
Zn2+ dependent	I		Sin3 coREST NuRD MiDAC	HDAC1	Belinostat, Chidamide, Entinostat, Mocetinostat, Panobinostat, Quisinostat, Romidepsin, SAHA/Vorinostat, TSA, Valproic Acid	Nucleus
				HDAC2	Belinostat, Chidamide, Entinostat, Mocetinostat, Panobinostat, Quisinostat, Romidepsin, SAHA/Vorinostat, TSA, Valproic Acid	
				HDAC3	Belinostat, Chidamide, Entinostat, Mocetinostat, Panobinostat, Quisinostat, SAHA/Vorinostat, TSA, Valproic Acid	
				HDAC8	Belinostat, Panobinostat, Quisinostat, SAHA/Vorinostat, TSA, Valproic Acid	
	II	IIa	N-CoR SMRT	HDAC4	Belinostat, Panobinostat, Quisinostat, Romidepsin, SAHA/Vorinostat, TSA, Valproic Acid	Cytoplasm/Nucleus
				HDAC5	Belinostat, Panobinostat, Quisinostat, SAHA/Vorinostat, TSA, Valproic Acid	
				HDAC7	Belinostat, Panobinostat, Quisinostat, SAHA/Vorinostat, TSA, Valproic Acid	
				HDAC9	Belinostat, Entinostat, Panobinostat, Quisinostat, SAHA/Vorinostat, TSA, Valproic Acid	
		IIb		HDAC6	Belinostat, Quisinostat, Romidepsin, SAHA/Vorinostat, TSA	Cytoplasm
				HDAC10	Belinostat, Chidamide, Quisinostat, SAHA/Vorinostat, TSA	
	IV		SMN	HDAC11	Mocetinostat, SAHA/Vorinostat	Cytoplasm/Nucleus
NAD+ dependent	III			SIRT 1-7	SAHA/Vorinostat	

NuRD, Nucleosome Remodeling and Deacetylating; MiDAC, mitotic deacetylase; N-CoR, nuclear receptor co-repressor; SMRT, silencing mediators for retinoic acid and thyroid hormone receptors; SMN, Survival of Motor Neurons.

This is primarily the result of the prevalence of fusion oncogenes, where the HDAC limits their function by genetic suppression. Furthermore, HDACi (TSA and vorinostat) were also able to downregulate key oncogenes associated with translocation events (17). The BCOR gene, a corepressor for the BCL6 protein that plays a crucial role in immune system cells has been found in up to 15% of cases of FN-RMS (18). This mutation is particularly susceptible to class I and II HDACi (17).

The decreased susceptibility of FN-RMS to HDACi compared remains unknown. Although FN-RMS usually contains a mutation in Ras signaling, other cancers with Ras mutations have shown vulnerability to HDACi. This includes non-small-cell lung carcinoma, colorectal carcinoma, and pancreatic carcinoma (19).

Clinical investigations of the HDACi RMS are challenging, because the differing biological and molecular subtypes can also differ the activity of specific HDACi and classes. However, past and ongoing trials suggest the potential for HDACi to mitigate the progression of RMS.

### Histone deacetylase classes and complexes

As noted previously, HDACs regulate transcription by removing acetylation on histones, which makes the chromatin less accessible to transcription factors. HDACs, however, do not perform DNA catalytic and localization activity in isolation. They require interactions with large transcriptional regulation complexes to confer precise DNA recruitment, remodeling, and co-repression (20). These interactions are outlined in **Table 2**.

Class I HDACs interact with Sin3, Co-REST, Nucleosome Remodeling and Deacetylating (NuRD) complex, and mitotic deacetylase (MiDac) complex (**Table 2**) (20, 21). Ultimately, these interactions are required for modulating catalytic activity and localization. The Sin3 complex serves an integral role in histone deacetylation and transcriptional repression (22). The Co-REST complex, upon associating with HDACs, functions as a chromatin remodeling complex, conferring repression (20, 23). NuRD is driven by protein subunits, including MBD2 and MBD3, to localize to DNA (24). When interacting with HDACs, the NuRD-HDAC complex can inhibit gene expression through the catalytic removal of acetyl marks on histones (24). MiDAC has been associated with a direct interaction with the catalytic portion of HDAC complexes to modulate target specification (21).

Interactions with silencing mediators for retinoic acid and thyroid hormone receptors (SMRT) and nuclear receptor co-repressor (N-CoR) are necessary factors for the activation of class III HDACs (**Table 2**) (25). Additionally, this class of HDACs involves an NAD+ cofactor that other HDAC classes do not. SMRT and NCoR recruit class III HDACs to the core of the complex. Upon binding with an inositol tetraphosphate molecule, the complex becomes stabilized and activated (26).

The N-CoR-SMRT-HDAC complex can also recruit HDAC Class IIa enzymes at the C-terminal of the HDAC polypeptide sequence (**Table 2**) (20). This protein complex does not enhance the catalytic activity of Class IIa HDACs but contributes to the recruitment of the larger transcriptional complex. Moreover, studies have found that this class of HDACs contain zinc-binding domains that regulate the structure and function of the protein (20, 27).

Class IV HDACs are not well understood and not well characterized. However, research has indicated that this class of HDACs typically interacts with the Survival of Motor Neurons (SMN) complex, which has important functions in small nuclear ribonucleoprotein (snRNP) assembly complexes (**Table 2**) (28).

The HDAC complexes can also be targeted by other transcription and binding factors including methyl-binding proteins, DNA methyltransferases, and histone methyltransferases. These large complexes give HDACs different targets and regulatory applications. Dysregulation of HDACs, therefore, confers epigenetic changes that could lead to oncogenesis. As such, aberrant localization may result from the interaction between fusion proteins that arise from chromosomal translocations. In ARMS, this manifests in the result of PAX-FOXO1 fusion proteins which work as transcription factors that promote tumor growth and development (29, 30).

Recent work has also revealed the effects of HDAC on core regulatory transcription factors (TFs) in FP-RMS. It was found that Class I HDACs are important for core regulatory transcription factor circuitry. As such, HDACi that inhibit Class I HDACs were investigated against other HDACi. In one study, HDACi activity on FP-RMS and FN-RMS was characterized. The results found that class I HDACi had the most prominent effect while Class IIa, Class IIb, and Class III HDACi had little anti-cancer effects (31, 32).

### Mechanisms of HDAC inhibitors on RMS

#### Pro-apoptotic effects of HDAC inhibitors on RMS

**Figure 1:**
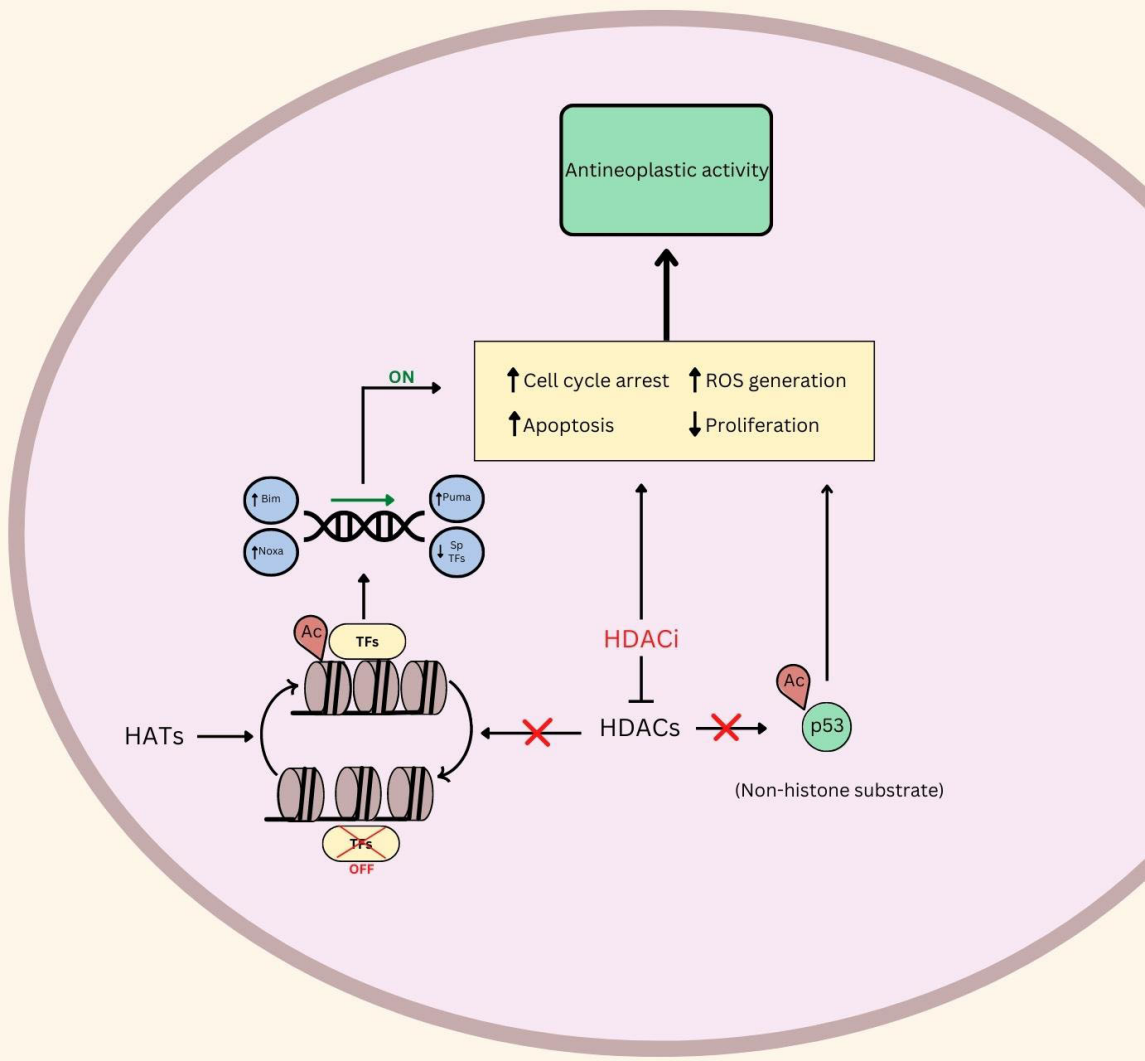
An illustration of the putative role of HDAC and HDACi in Rhabdomyosarcoma. ⟂ indicates inhibition, ↑ indicates upregulation or promotion, ↓ indicates down regulation. Ac, Acetylated; TFs, Transcription Factors; ROS, Reactive Oxygen Species; HDACs, Histone Deacetylases; HDACi, Histone Deacetylase Inhibitors; HATs, Histone Acetyl Transferases.

RMS, like many cancer types, can proliferate uncontrollably due to their ability to circumvent programmed cell death. The research discussed in this review illustrates that HDACi induce apoptosis in RMS through various mechanisms (**Figure 1**).

Several studies indicate that suberoylanilide hydroxamic acid (SAHA) induced apoptosis in RMS cell lines (30, 33). In an analysis of SAHA, a TUNEL assay elucidated that HDACi treatment led to an increase in apoptosis in 4 out of 5 RMS cell lines (30). However, Rh30 cells showed no significant changes in apoptosis, despite seeing increases in apoptosis in Rh41 (both are ARMS cell lines). The molecular underpinnings behind the distinction in the Rh30 cell lines are yet to be elucidated. Ultimately, however, these studies were able to associate the increase in apoptosis with phosphorylation of Histone H2AX, which marks DNA damage, in the majority of RMS cell lines (30).

Another investigational HDACi (OBP-801) increased apoptosis levels post-treatment in 8 RMS cell lines (34). The group used immunostaining to identify that downregulation of survivin and chromosome misalignment, secondary to treatment, were the cause of apoptosis, mediated by the introduction of the HDACi. Furthermore, TSA also induced apoptosis at sufficiently high doses (500>nM) according to Annexin V staining in Rh30 (ARMS) and RD (ERMS) cell lines (35). Another HDACi, JNJ-26481585, upregulates Bim, Puma, and Noxa proteins in Rh30 and RD cells, which promotes programmed cell death (**Figure 1**) (36). The putative role of HDACi in promoting apoptosis and programmed cell death in RMS makes this class of agents exciting for further clinical exploration.

#### HDAC inhibitor-induced modulation of oxidative stress

A recent study found that ROS were present in higher concentrations in RMS in comparison to other cancer types, and there is accumulating evidence that RMS may be particularly susceptible to treatments that increase ROS sensitivit (37, 38). There is also evidence that attests to the potency of HDACi in inhibiting RMS progression through ROS-dependent mechanisms (4, 39). However, more information regarding the role of HDACi in sensitizing RMS cells to ROS damage needs to be further explored to gain a more comprehensive understanding of the reliance of HDACi on ROS.

There is a growing body of evidence indicating that treatment using HDACi leads to an increase in reactive oxygen species (ROS) (4). Subsequently, it was shown that treatment using HDACi (panobinostat and vorinostat) led to notable increases in various apoptotic markers, including annexin V, and cleavage of PARP and caspase 3 in RD and Rh30 cells. Notably, the apoptotic effects were reversed after treatment with antioxidants. As such, there is evidence indicating that apoptosis as a result of HDACi may be attributed to an increase in ROS levels (4).

One such study linking RMS oncogenic behavior with ROS demonstrated that the inhibition of RMS cell growth and proliferation by using panobinostat or vorinostat was reversed by co-treatment with glutathione, which prevents ROS-related damage (4). This study also found that the HDAC inhibitor treatments significantly increased ROS in xenografts from RMS patients. Furthermore, the researchers illustrated that HDACi led to the downregulation of Sp1, Sp3, and Sp4 transcription factors, similar to the effects of hydrogen peroxide - a potent source of ROS. Genetic analysis led them to conclude that Sp TFs were inhibited as a result of ROS-mediated cMyc repression (**Figure 1**). These results, coupled with their observations that antioxidants mitigated the downregulation and antineoplastic activities of HDACi, led them to conclude that HDACi rely, at least in part, on ROS induction. Panobinostat and vorinostat were also investigated in clinical trials, discussed below (**Table 1**) (4).

Another study investigating the role of HDACi in increasing oxidative stress in RMS indicated that entinostat caused increased levels of intracellular ROS in xenograft mouse models (12). Although this study also highlighted cessation of tumor progression as a result of treatment using entinostat, it did not elucidate if the antineoplastic effects were solely attributed to the increase in intracellular ROS levels. An additional evaluation of entinostat found that upon treatment, RMS cells experienced rapid and sustained onset of high levels of ROS for the 12 hours of the experiment (39). According to the same study, these results were more sustained than ROS generation as a result of radiation therapy alone. These results were later validated by a subsequent study, which showed that belinostat works synergistically with radiation therapy by preventing the phosphorylation of extracellular signal-regulated kinases (ERKs) that are known to confer radioresistance (40).

#### Cell cycle regulatory capabilities of HDAC inhibitors

Through the inhibition of HDACs, treatments can induce cell cycle arrest at various points in interphase prior to cell replication (**Figure 1**). The process of cell cycle arrest allows for a stop in the proliferation process that can serve as a form of treatment for RMS. A former study into the application of inducing cell cycle arrest in human cervix carcinoma HeLa cells found the process to occur during the G1 phase, ultimately supporting the idea that the progression of cell duplication can be halted, and histone acetylation is involved in the process of cell cycle control. Specifically, inhibiting HDAC causes the chromatin of the cell to become hyperacetylated (41).

Trichostatin A (TSA) was shown to induce G1 arrest by the induction of the p21 gene. As such, studies have indicated that the pro-apoptotic, antineoplastic activity of HDACi can be modulated by cell cycle regulation (42). G1 arrest can also be driven by the HDAC inhibitor apigenin which induces apoptosis and supports the expression of the Fas/Fas ligand, a molecule that can bind to the Fas receptor and initiate a chain of reactions to cell cycle arrest in CD4+ T cells (43).

Checkpoints between G1 and S are important for preparing the cell for DNA replication and eventual mitosis. A study conducted with various HDACs found that defects in HDAC null cells removed the ability for double-stranded breaks in the DNA to be repaired, resulting in S phase-associated DNA damage and forcing the cell into S phase arrest after the checkpoint is initiated (42). Furthermore, the HDAC inhibitor, SAHA, plays a follow-up role with the p21 tumor suppressor gene, pushing the cell into S-phase cell cycle arrest due to activation of the DNA damage response pathway (30).

Finally, G2 arrest is driven by G2 cyclin inactivation. Using a yeast G2 checkpoint model, it was found that belinostat is able to activate the G2 checkpoint in the cell cycle, which presents the opportunity for cell cycle arrest and potential apoptosis (38, 40, 44). This checkpoint is usually defective in tumor cells, which can contribute to uncontrolled proliferation. Specifically, HDACi induce the cell into a G2 phase checkpoint, which has been found to be defective in tumor cells (41).

#### Synergistic potential of HDAC inhibitors (with chemotherapy, radiation therapy, and small molecules)

Cancer is a multifaceted disease with various epigenetic, genetic, and pathological factors. As such, multiple combinatorial treatments are often required. Synergistic approaches to cancer treatment utilize various mechanisms to target the disease, minimizing the risk of adverse side effects while increasing treatment efficacy by encompassing a wide range of targets. RMS is no exception to this paradigm, where the treatment paradigm consists of combination chemotherapies, radiation in many instances, and often surgery when feasible. Thus, it is important to analyze the effects of HDACi in combination with standard-of-care treatments and other novel approaches. In this review, we have identified studies that analyze the anti-cancer effect of HDACi in combination with other drugs and chemotherapeutic approaches.

Standard-of-care chemotherapeutics are well-studied with a history of providing high response rates and overall cures for patients with RMS. So, it is unlikely that a novel treatment will replace the current treatment paradigm. However, for a subset of RMS patients with translocation positive, metastatic, refractory and/or recurrent disease the outcomes are dismal, and survival is the exception rather than the rule. Addition of new targeted agents to the current standard RMS armamentarium are ongoing. Studies have investigated the effect of HDACi when combined with current treatments. HDACi in combination with cisplatin also demonstrated additive anti-cancer effects (45). In another study, SAHA in combination with treatments currently used in clinics (Doxorubicin, Etoposide, Vincristine and Cyclophosphamide) led to significant increases in apoptosis in RMS cell lines, as indicated by a combination index (CI) (46, 47).

Many preclinical studies have indicated that HDACi synergize with radiation therapy to amplify anti-cancer effects on RMS (39, 40, 48, 49). In an *in vitro* and *in vivo* study, MS-275 (entinostat) was shown to radio-sensitize RH30 (FP) cell lines (39). Interestingly, however, the results from these studies indicate that RD (FN) cell lines did not experience significant radiosensitization (39, 49). This was marked by expression of PI3K, Akt, mTOR, and MAPK, which are genes involved in chemoresistance. However, there is an indication of a decrease in c-Myc expression in RD cells, potentially indicating slower proliferation rates of RD cells due to radiation combined with entinostat (39). Similar studies using other HDACi, such as SAHA and PXD-101 (belinostat), indicated that HDACi decrease clonogenic survival when combined with radiation therapy to a greater extent than either of the two treatments on their own (40, 49). The results from these studies indicate a statistically significant increase in predisposing RMS cell lines to respond to radiation therapy. A clinical trial investigating the clinical potential of belinostat is discussed in the following section of this review (3.4, **Table 1**).

As mentioned previously, HDACi exhibit pro-apoptotic effects on cancer cells. As such, several studies have investigated the combined effects of HDACi with BCL-2 inhibitors, which are known anti-apoptotic proteins (50–52). These studies have demonstrated a significant reduction in RMS cell viability upon co-treatment with HDACi and BCL-2 inhibitors with more efficiency than either candidate used in isolation.

As discussed earlier, valproic acid, in combination with midostaurin, was shown to downregulate PAX-FOXO1 activity and ARMS proliferation. The investigation into this drug also showed that a combination of an HDACi with midostaurin led to a more potent effect than a midostaurin-only treatment (16). Thus, HDACi may improve the efficacy of other mechanistic approaches. Valproic acid was involved in a clinical investigation for its use in treating sarcomas (3.4, **Table 1**).

Synergistic treatments using HDACi and other agents not only improves the efficacy of treatments, but mitigates potential risks associated with broad epigenetic modifiers. One study found that Ezrin, a protein linker that regulates complexes associated with the membrane and cytoskeleton, was in high levels of metastatic RMS and correlated with hyperacetylation (45). As such, despite the demonstrated benefits of HDACi, there is concern that subsequent hyperacetylation may increase the likelihood of metastasis of the cancer upon treatment. In order to mitigate the undesirable “off-target” effects, the same study showed that a combinatorial approach using Ezrin-specific shRNA with TSA prevented Ezrin’s upregulation while maintaining the antineoplastic activity of TSA. Thus, combinatorial approaches not only confer synergistic benefits but may be necessary to mitigate adverse side effects. Taken together, current research attests to the potential of HDACi to be used as an adjunct therapy with novel and standard-of-care approaches and chemotherapeutics.

### Evaluation of HDACi and clinical investigation statuses

Currently, two active trials are studying the efficacy of HDACi for RMS patients (**Table 1**). The first is a Phase 1 trial studying the effects of the HDACi, vorinostat (Merck & Co., Inc., New Jersey, USA), and the combination of vorinostat, vincristine, irinotecan, and temozolomide on patients with relapsed or refractory solid tumors, like RMS (NCT04308330). The second is a Phase 1 trial investigating the effects and best dose of a combination of Vinorelbine and the HDACi, vocetinostat (Mirati Therapeutics, California, USA) (NCT04299113) on a schedule of 40mg, 3 days a week, in children, adolescents, young adults with refractory and/or recurrent RMS (**Table 1**). Preliminary results of this early phase trial have been presented at American Society of Clinical Oncology Annual Meeting (Chicago, US, June 2022) and are encouraging for early activity in both translocation positive and negative RMS. The most common dose-limiting toxicities observed were grade 5 neutropenia and anemia, and grade 4 nausea. Early results have shown that out of 8 subjects, 2 subjects had stable disease and 1 subject had progressive disease for a clinical benefit rate of 86%. (53) Overall, early data supports an acceptable toxicity profile, and activity of HDAC Class 1 therapeutics in the RMS anti-cancer armamentarium.

Entinostat (Syndax Pharmaceuticals Inc., Massachusetts, USA), or MS-275, has been studied in combination with other therapies for RMS treatment. Preclinical *in vitro* and *in vivo* work has investigated the effects of entinostat as a single agent and in combination with other therapeutics in RMS (31, 39, 44, 51, 54–58). Synergistic treatment utilizing entinostat and radiation therapy *in vivo* led to a complete prevention of RH30 (FP-ARMS) cell growth *via* G2 growth phase cell arrest (52). Entinostat has been explored in human subjects with relapsed or refractory solid tumors in a phase 1 clinical trial (NCT02780804).

Panobinostat (Secura Bio, Inc., Nevada, USA) has not been often utilized in RMS research. However, this HDACi was more efficient in inducing mitochondrial apoptosis in conjunction with BET protein inhibitors. By shifting the ratio of pro- and antiapoptotic BCL-2 proteins in favor of apoptosis, these drugs displayed anticancer effects in RMS cells (51). Additionally, when panobinostat was combined with another HDACi, vorinostat, the treatment inhibited RMS growth *in vivo via* cancer cell apoptosis (4). In 2015, Panobinostat was FDA-approved for treating multiple myeloma in patients whose cancer has progressed after treatment with at least two prior standard therapies. Panobinostat and Vorinostat have been involved in clinical trials for the treatment of sarcomas (3.4, **Table 1**).

The HDACi OBP-801 (Oncolys BioPharma Inc., Japan) is also not frequently used in investigations for RMS treatments. However, a study has revealed that OBP-801 targets p21, the cyclin dependent kinase inhibitor that regulates cell proliferation, to arrest the cell cycle of RMS cells. After 24 hours of exposure, OBP-801 inhibited RMS cell line growth and induced apoptosis and DNA damage (34). OBP has not been approved by the FDA for any treatments, but in 2014 it received an investigational new drug approval to conduct Phase 1 clinical trials as a novel epigenetic cancer drug.

When investigated as a treatment for RMS, vorinostat, or SAHA, successfully inhibited the progression of ERMS and ARMS cells by inducing apoptosis and activating a DNA damage response (30). In ERMS cells, tumor growth was inhibited by inducing myogenic differentiation, thus inhibiting migration capacity and reducing the proliferative capacities of the cancer cells (17). vorinostat works synergistically with other HDACi, such as pyroxamide, and chemotherapeutics like cisplatin to suppress RMS growth and progression. However, there is no evidence vorinostat significantly increases radiation-induced apoptosis in RMS cells (49). Vorinostat, became the first HDACi approved by the FDA in 2006 for the treatment of cutaneous manifestations of cutaneous T-cell lymphoma (CTCL) in patients with progressive, persistent, or recurrent disease on or following two systemic therapies. Vorinostat was explored in several completed solid tumor and sarcoma clinical trials. Investigated for the treatment of metastatic soft tissue sarcoma, three cycles of vorinostat, 400mg q.d., displayed disease stabilization in 23% patients, and long-lasting disease stabilization for up to ten cycles in 15% of patients (NCT00918489). The most common grade ≥ 3 toxicities observed included hematological toxicity, gastrointestinal disorders, fatigue, musculoskeletal pain, and pneumonia (59). As mentioned, currently, an open and accruing trial is investigating vorinostat in combination with chemotherapies for the treatment of RMS (NCT04308330, **Table 1**).

TSA is a HDACi that induces cell apoptosis and myogenic differentiation, inhibiting migration and proliferation capabilities of RMS cells (17, 35). In an attempt to improve immunotherapy receptivity, a study revealed a combined therapy of TSA and DNA methyltransferase restores previously silenced CIITA expression in ARMS cells. As CIITA regulates expression of MHC class I proteins, TSA may improve the antigen presentation of RMS cells (60). Overexpression of plakoglobin, a component of cell-cell adherent junctions, has tumor suppressing properties. However, a study revealed plakoglobin expression was absent in ARMS cells. An intervention 5AzadC, a DNA demethylating agent, and TSA restored plakoglobin expression in ARMS cells (61). Currently, TSA is not approved by the FDA, but is under active clinical investigation across various indications.

Quisinostat (Janssen Pharmaceuticals, Belgium), or JNJ-26481585, activates the mitochondrial apoptosis pathway by triggering the Bax/Bak complex, which activates enzyme caspase-9 (36). Studies showed that quisinostat works synergistically with treatments, such as LSD1 inhibitors or a BH3 mimetic, ABT-199, to activate apoptosis pathways in RMS cells (50, 62). There are no current clinical trials investigating quisinostat for treatment of RMS.

The HDACi valproic acid (Abbott Laboratories Inc., Illinois, USA) displays antitumor effects by reactivating the p21 gene, which regulates cell proliferation by inhibiting the cell (46). Additionally, when combined with the small molecule inhibitor PKC412, valproic acid induced apoptosis and suppressed growth of RMS (16). In 2008, valproic acid was approved by the FDA for the treatment of bipolar disorder, seizures, and migraine headaches.

While hundreds of studies are being conducted with HDACi for other conditions, and some have been approved by the FDA, currently only two trials are actively investigating HDACi as treatment for RMS. Past studies have revealed the cellar mechanisms and the anticancer benefits of HDACi treatments for RMS. Clearly further exploration of HDACi in RMS is warranted.

### Potential side effects and toxicity of HDACi

Given the focus on the pediatric population for the potential use of HDACi as a treatment option, analyzing their side effects and toxicity is important. This analysis of the HDACi side effects and toxicity profile is not specific to the treatment of RMS. Similar to other therapeutics, there are reported adverse effects associated with the use of HDACi. Through the analysis of vorinostat and panobinostat for treating multiple myeloma, adverse effects have been seen both hematologically and non-hematologically. Anemia, neutropenia, and thrombocytopenia were the most common hematological ones and fatigue/asthenia, diarrhea, and nausea were the most common non-hematological ones, with all found in similar frequencies for both HDACi (63). For the treatment of metastatic neuroendocrine tumors, cardiac toxicity was observed, with a specific concern of ventricular arrhythmias following the use of HDACi (64). With using mocetinostat for the treatment of relapsed classical Hodgkin’s lymphoma, the most common adverse effects include myelosuppression, fatigue, and pneumonia (65). HDAC inhibitor-induced thrombocytopenia was also observed through the use of panobinostat, which has played a role in determining dose limits for HDACi (66).

## Conclusions

Research on RMS has made considerable progress in alleviating the burden, increase life-expectancy, and decreasing morbidity in patients. Novel advancements are constantly being pushed to target RMS and provide adjunct approaches. One particular approach that has recently garnered attention in the field is to target HDACs with HDACi which has been proven effective in other cancer types. As such, our review summarizes the current research in the field. This review finds that HDACi are a potent inhibitor of RMS proliferation, as suggested by *in vitro* and *in vivo* findings that highlight the mechanisms, benefits, and efficacy of the treatment modality. More specifically, our re-view finds that HDACi successfully modulate oxidative stress, inhibit cell cycle progression, induce pro-apoptotic effect, and work synergistically with novel and standard of care treatments to evoke anti-cancer effects in RMS. Future studies should aim to elucidate the populations of RMS patients including RMS molecular subtypes, who would most benefit from the treatment as well as if there are any notable differences between the drugs currently under investigation.

## Current limitations and future directions

As discussed previously, HDACi have been known to cause side-effects in the clinic. Although there is extensive research documenting the tolerability and efficacy of HDACi on hematologic cancers, historically, HDACi have been ineffective against solid tumors (67–69). In addition to synergistic treatments that have been shown to increase the efficacy of HDACi, more research should be conducted into the specific roles in core regulatory TFs for RMS and identify the distinguishing features between efficacy in FP-RMS compared to FN-RMS.

## Author contributions

Conceptualization, OS; validation, NF and AS; formal analysis, OS, CS, AK, and RP; investigation, OS, CS, AK, and RP; resources, OS, CS, AK, and RP; data curation, OS, CS, AK, and RP; writing original draft preparation, OS, CS, AK, and RP; writing review and editing, NF and AS; visualization, OS; supervision, NF and AS; project administration, NF and AS; funding acquisition, NF. All authors have read and agreed to the published version of the manuscript.
